# Supplementary figures and images for: Data-driven optimization of diet formulation to enhance survival and growth in Japanese Eel (Anguilla japonica) larvae
Source: PLoS One. 2026 Feb 12;21(2):e0342983. doi: 10.1371/journal.pone.0342983 (PMC12900294; doi:10.1371/journal.pone.0342983)

6-40 dph

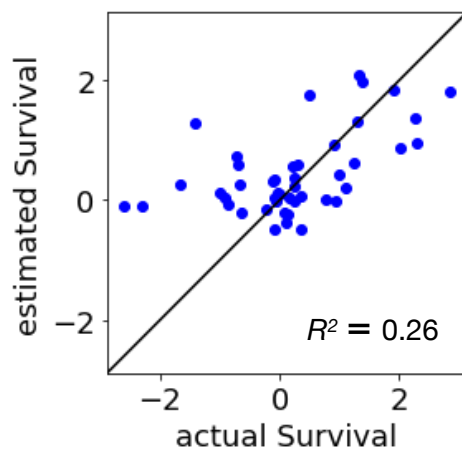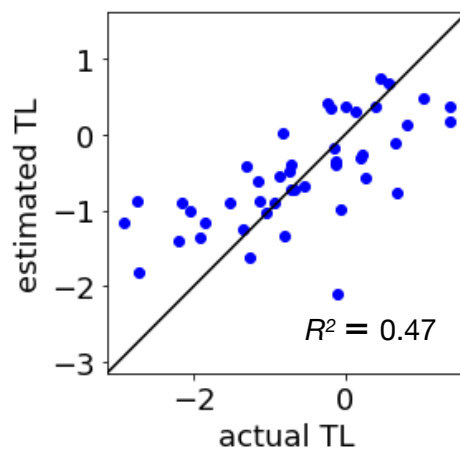

41-80 dph

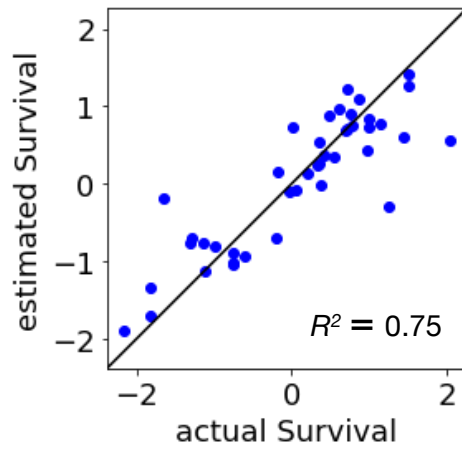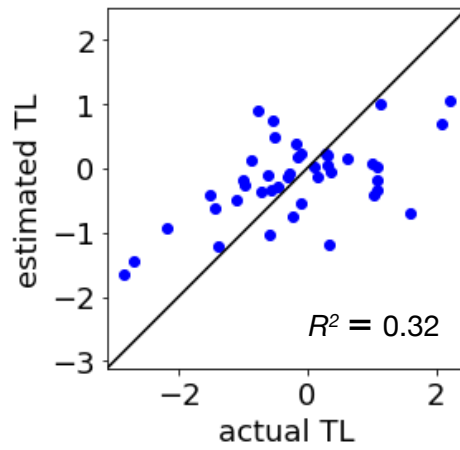

Supplement: S1 Fig — Each panel shows the relationship between predicted and observed standardized z-scores for survival rate or mean total length (TL) during two developmental periods (6–40 dph and 41–80 dph). Predictions were obtained using 10-fold cross-validation. (PDF) [file pone.0342983.s001.pdf]

## 6-40 dph

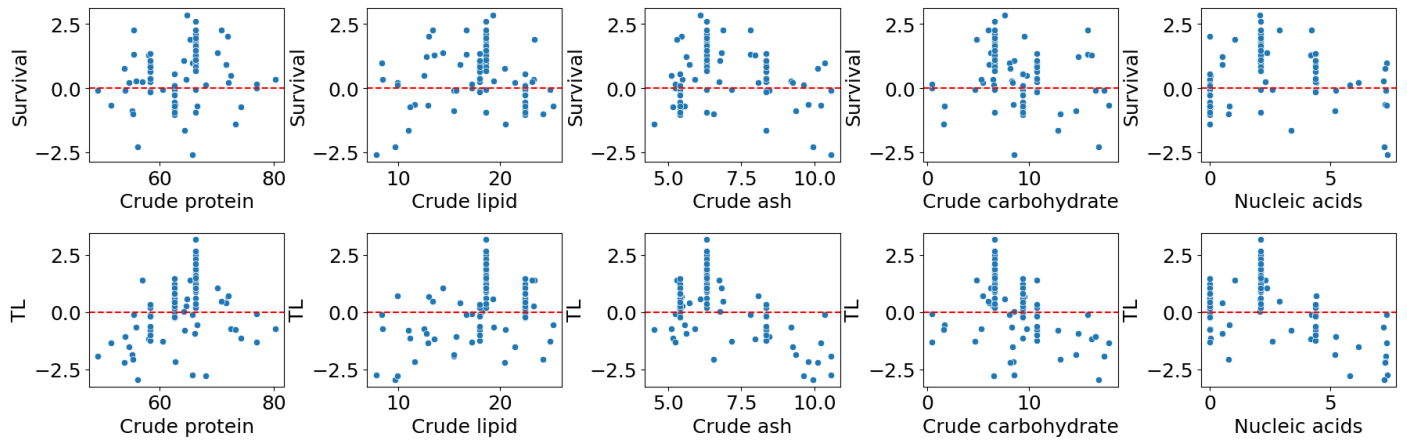

## 41-80 dph

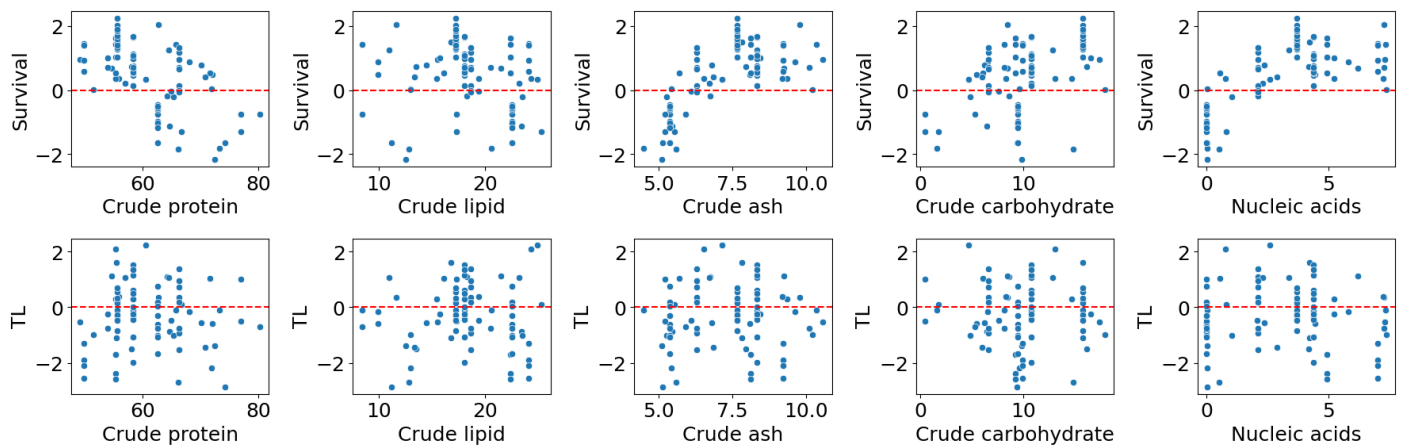

Supplement: S2 Fig — Each panel shows the relationship between the percentage of crude protein, crude lipid, crude ash, crude carbohydrate, or nucleic acids in the diet (x-axis) and the standardized objective variables (y-axis; survival rate or mean TL) for each diet. Results are shown separately for the two developmental periods (6–40 dph and 41–80 dph). (PDF) [file pone.0342983.s002.pdf]

6-40 dph

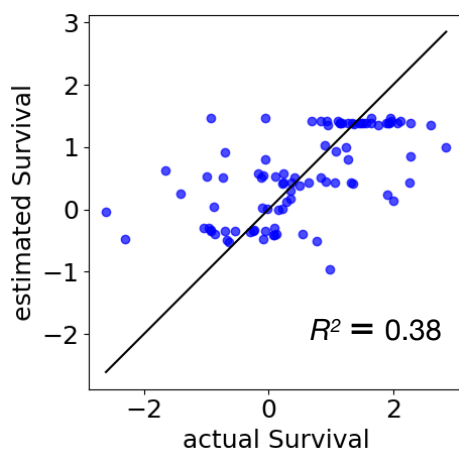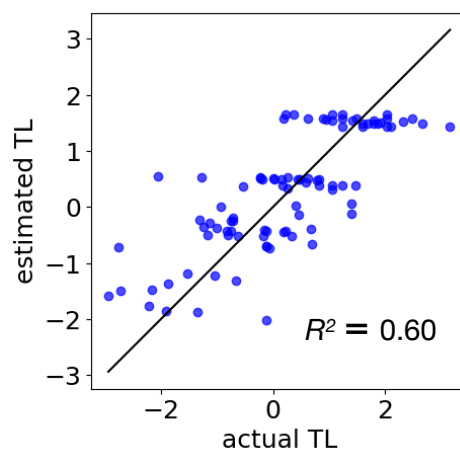

41-80 dph

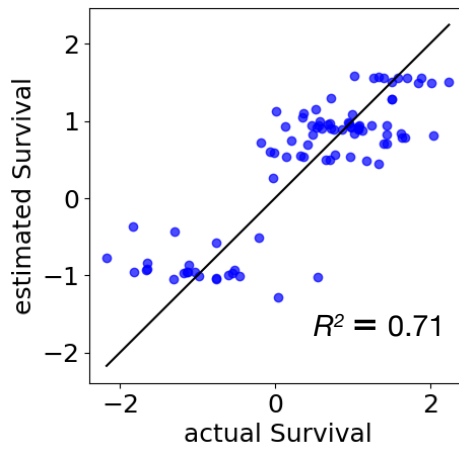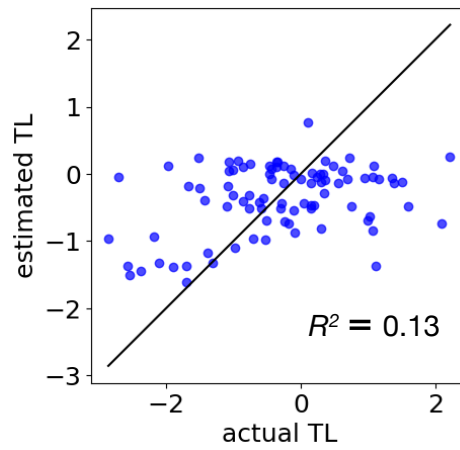

Supplement: S3 Fig — Each panel shows the relationship between predicted and observed standardized z-scores for survival rate or mean total length (TL) during two developmental periods (6–40 dph and 41–80 dph). Predictions were obtained using 10-fold cross-validation. (PDF) [file pone.0342983.s003.pdf]
